# Reliability of a new analysis to compute time to stabilization following a single leg drop jump landing in children

**DOI:** 10.1371/journal.pone.0212124

**Published:** 2019-02-12

**Authors:** Xavier García-Massó, Jiri Skypala, Daniel Jandacka, Isaac Estevan

**Affiliations:** 1 HUMAG Research Group, Department of Teaching of Music, Visual and Corporal Expression, University of Valencia, Valencia, Spain; 2 Department of Human Movement Studies, Human Motion Diagnostic Centre, University of Ostrava, Ostrava, Czech Republic; 3 AFIPS Research Group, Department of Teaching of Music, Visual and Corporal Expression, University of Valencia, Valencia, Spain; University of Innsbruck, AUSTRIA

## Abstract

Although a number of different methods have been proposed to assess the time to stabilization (TTS), none is reliable in every axis and no tests of this type have been carried out on children. The purpose of this study was thus to develop a new computational method to obtain TTS using a time-scale (frequency) approach [i.e. continuous wavelet transformation (WAV)] in children. Thirty normally-developed children (mean age 10.16 years, *SD* = 1.52) participated in the study. Every participant performed 30 single-leg drop jump landings with the dominant lower limb (barefoot) on a force plate from three different heights (15cm, 20cm and 25cm). Five signals were used to compute the TTS: i) Raw, ii) Root mean squared, iii) Sequential average processing, iv) the fitting curve of the signal using an unbounded third order polynomial fit, and v) WAV. The reliability of the TTS was determined by computing both the Intraclass Correlation Coefficient (ICC) and the Standard Error of the Measurement (SEM).In the antero-posterior and vertical axes, the values obtained with the WAV signal from all heights were similar to those obtained by raw, root mean squared and sequential average processing. The values obtained for the medio-lateral axis were relatively small. This WAV provided substantial-to-good ICC values and low SEM for almost all the axes and heights. The results of the current study thus suggest the WAV method could be used to compute overall TTS when studying children’s dynamic postural stability.

## Introduction

Dynamic postural stability has up to now been considered as a capacity associated with impaired neuromuscular conditions and potential performance in sports [1–5]. Due to the lower limb functional asymmetry linked to health, maturation and aging, postural stability has mainly been studied in older adults [6]. However, according to the theory of a developmental landscape in which more consistent coordination patterns are obtained with increasing age [7,8], it is important to analyze and understand the mechanisms of dynamic postural stability in different age-related groups, such as prepubescent children, who seem to have poorer neuromotor control than adolescents [9]. Due to this limited motor development, young children’s coordination patterns are unstable and characterized by high movement variability [7].

Because of the dynamic and instantaneous variation states in balance [6], the single leg drop jump landing is often used to measure dynamic postural stability [10]. In line with other vertical jumps, such as the countermovement jump [7], the single leg drop jump landing is a discrete task with no continuous cyclic pattern and has a distinct start and end. As this task is not usually taught except in jump-specific sports [7], the individual developmental level is reflected in one’s ability to maintain balance. Postural stability during a single leg drop jump landing is often evaluated by the analysis of the ground reaction forces (GRF) and related variables [e.g., center of pressure, time to stabilization (TTS), etc.] during the landing.

TTS is one of the most common outcome measures used to characterize GRF during the single leg drop jump landing [11]. However, several of the parameters involved in its calculation can affect the values obtained. Although the effect of the time signal, sample frequency and filtering settings has previously been pointed out [12], there are also other factors that should be considered, such as: i) axis of the ground reaction force, ii) signal processing, iii) threshold and iv) height of the jump.

TTS can be computed in the three GRF axes [i.e., vertical (V), antero-posterior (AP) and medio-lateral (ML)], although different values may be obtained from each one. Liu & Heise [12] found considerable differences between the TTS of drop jump in different directions, while Colby, Hintermeister, Torry and Steadman [13] reported that TTS varied according to the GRF axis.

Fransz et al. [12]conducted a study on adults in which four signal processes were applied to GRF data: i) no processing at all (RAW), ii) a root mean square (RMS) average with a moving window, iii) sequential average processing (SA), and iv) the fitting curve of the signal using an unbounded third order polynomial fit (TOP). The TTS values obtained by these methods ranged from 0.11 to 6.6 s. In a similar study on adolescents, Fransz, Huurnink, de Boode, Kingma and van Dieën [14,15] showed that both the SA and TOP methods provided sufficiently reliable TTS values for both the V and the AP-axis, but not for the ML. However, the processing signal applied did not provide reliable values for the ML-axis either in adults or adolescents.

The threshold method most frequently used to obtain TTS is the overall series mean ± standard deviation (SD), in which the TTS range is between 1.3 and 6.6 s [12]. When Fransz et al. [14,15] aimed to determine the effect of the number of SDs used as threshold on TTS reliability, they found that the value is highly dependent on the threshold used and that none of the processed signal—threshold combinations provided a reliable threshold value. This indicates the need for studies to obtain the ‘best’ threshold level in child populations by means of automatic computational methods.

The height of the jump may also have a strong influence on TTS reliability and values. When jumps are performed from considerable heights, both adults and children appear to use landing strategies to attenuate GRFs [16]. The heights of single leg drop jumps vary from 15 to 50 cm, according to the population studied (i.e. adults or children) [10,16], with a height of 20 cm or more giving no benefit to the children’s performance, partially attributed to their immature technique [17], indicating the need for studies on the effect of jump height on TTS reliability.

Since no study has so far found a reliable method of computing TTS in all three axes, our main aim was to develop a new computational method of obtaining TTS using a time-scale (frequency) approach on children. The reliability of this method was compared to that of the previously used methods for jumps from different heights, GRF directions and thresholds. Moreover, the sensibility of the different methods was established comparing the TTS among three different jumps heights.

## Material & methods

### Participants

First of all, sample size calculation methods for reliability studies were applied in order to determine the amount of participants needed in this study [18,19]. Using the lowest ICC value (i.e., 0.5) from Fransz et al. [15], an alpha level of 0.05 and a power of 0.9 the amount of children recommended was thirty. Thus, a non-probabilistic sampling method was used to recruit thirty children (14 boys and 16 girls), who voluntarily participated in this study (mean age, weight, and height were 10.16 years, *SD* = 1.52; 33.79 kg, *SD* = 10.93; and 1.39 m, *SD* = .11, respectively). Before participating in the tests, the parents of the participants gave their written informed consent and confirmed none of the children had a history of injury within the six months before the tests were carried out. Although girls appear to mature slightly earlier than boys, during prepubescence (6–13 years old) there are no differences between boys and girls in balance control when performing single-leg balance and drop jumps [9], so both girls and boys were considered as a single cohort in this study. The study procedure was approved by the Ethics Committee of the University of Valencia (H1446557620395).

### Experimental design

The participants landed with the dominant lower limb (barefoot) on a force plate (Kistler 9286AA, Switzerland) from three different heights (15cm, 20cm and 25cm) with their hands on their hips. After landing they were encouraged to maintain single-leg balance for 10 s. Ground reaction force data were collected at 240 Hz. Lower limb dominancy was established by asking the children to kick a ball three times as hard as possible; the leg used was considered as the dominant one (27 were right-footed and 3 left-footed). The trials were counterbalanced so that all the participants did not complete the tests in the same order.

The children were guided by a research assistant to warm up individually following a narrative story, including: lower limb joint mobility, jogging, lower limb stretching and small jumps, plus a specific warm-up that consisted of six trials–two single-leg landings from each height–to familiarize themselves with the landing process (total warm-up time 10 min approximately). Throughout the test, each participant was asked to step from the corresponding height with the landing leg on the force plates from a metal platform behind the force plate. The free leg was kept in the rear with the knee flexed 90° approximately. The trials were video recorded to reject those which: i) started jumping instead of stepping; ii) the knee of the free leg was not flexed around 90°; iii) the free leg touched the floor during the required 10 s balance period.

The participants received the standard instruction to land on their dominant leg and keep their balance while remaining as still as possible for 10 s after landing. Each trial started preferably between 1–3 s after the research assistant gave the appropriate signal and a rest interval was allowed between trials of 1–2 min. The children performed a total of 30 trials, 10 from each height.

### Data analysis

Data from the force platform were processed using Visual 3D software (C-motion, USA). The GRF was measured in each of three planes (the sagittal, *AP-axis*; the lateral, *ML-axis*; and the vertical component, *V-axis*).

GRF signals were filtered using a bidirectional Butterworth low-pass filter with a cut-off frequency of 12 Hz, after which the mean body weight value from 5 to 10 s was subtracted and the signals were rectified. Following the signal processing for proposed previously in other populations [11,14,15], the same four signals were used to compute the TTS: i) RAW, ii) RMS, calculated with a moving (1/240 s per step) windows of 250 ms, iii) SA was computed at each time point as the mean value of this and previous (not subsequent) data points, and iv) TOP of the raw signal starting at the maximum GRF point using the least-squares method.

**Fig 1:**
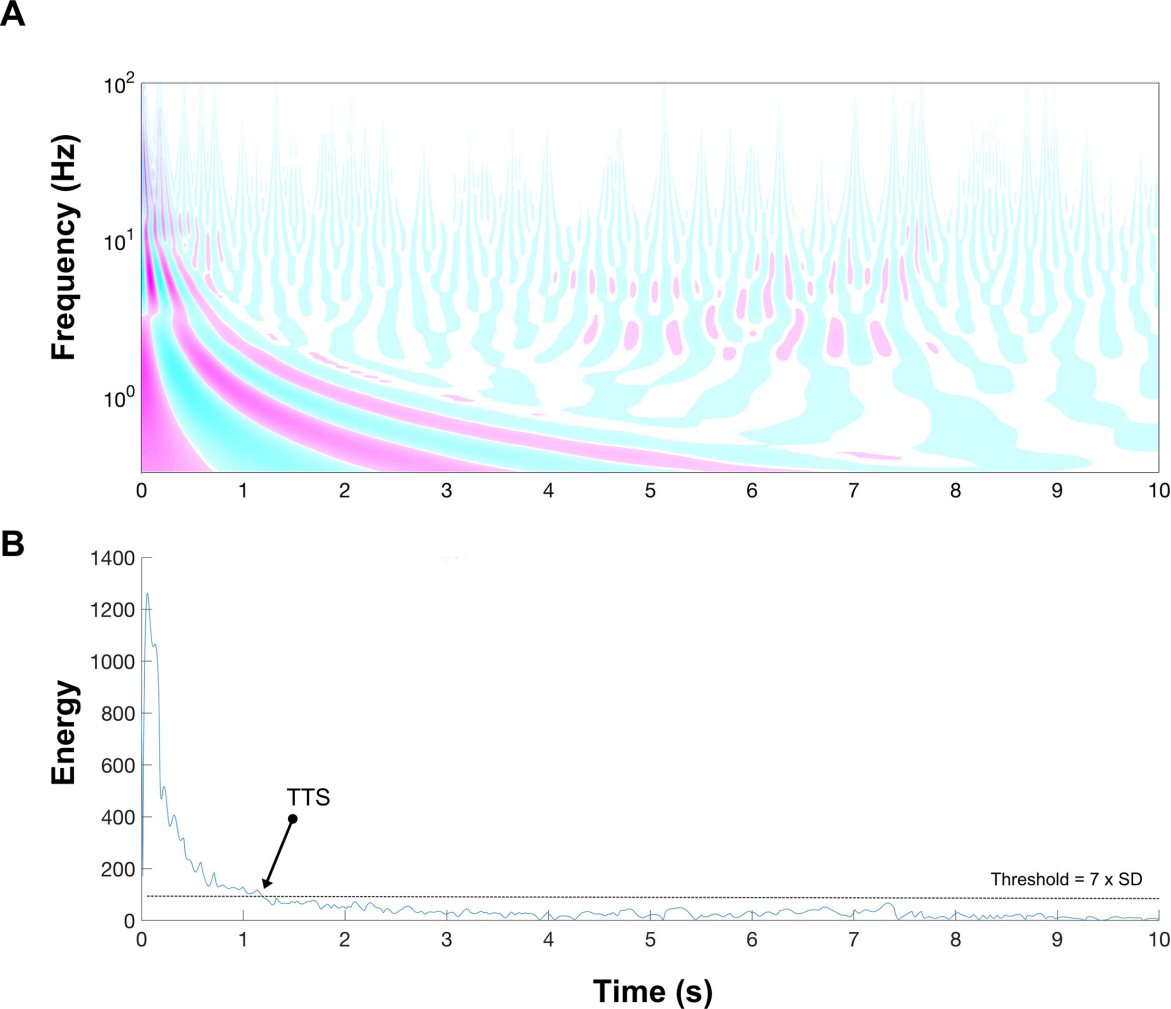
Example of a continuous wavelet transformation analysis performed to compute time to stabilization. A: continuous wavelet transformation surface. B: energy of the wavelet surface at each time point. TTS = time to stabilization; SD = standard deviation.

Since the previous analyses were based on time domain signals, we aimed to change the perspective of the problem by exploring the signals in the frequency domain using a time-scale (frequency) approach [i.e., continuous wavelet transformation (WAV)]. Similar processes have been developed previously to detect important events in different kinds of digital signals [20,21]. A Morse wavelet mother was used and the range of frequencies explored was from 0 to 100 Hz. Fig 1A shows a mesh plot of the WAV. The highest energy content can be seen to occur on landing. The wavelet energy was obtained at each signal point in the time axis by integrating the energy of the continuous wavelet in frequency (Fig 1B). This signal was used to compute the TTS.

The influence of the threshold on TTS reliability was determined by computing n-times the *SD* during the last 5 s of each signal. The TTS was then obtained by using a wide range of thresholds (between 1 to 160 SDs, with steps of 1 SD) at the point after the impact in which the signal (i.e. RAW, RMS, SA, TOP or WAV) crossed the threshold and remained under it for at least 0.5 s [14,22].

The effects of three factors on TTS and its reliability were also determined: i) the height of the jump (15, 20 and 25 cm), ii) the axis of the GRF (V, AP and ML), and iii) the signal used to compute the TTS (RAW, RMS, SA, TOP and WAV).

The time to body weight was calculated as the point at which the zero-mean RAW signal of the vertical GRF first passed zero after the impact force [14]. The mean time to body weight for all the signals of each of the methods evaluated was then calculated. Some of the extreme values of the TTS could not be computed [15] at low and high thresholds because they were lower than the time to body weight (for higher thresholds) or longer than the signal length (lower thresholds). In these cases, the further analyses were performed by discarding the cases in which TTS could not be calculated. The parts per unit of unusable trials were therefore determined for each of the forty-five conditions [i.e., combinations of signal processing (x5), GRF-axis (x3) and height (x3)] explored to compute TTS.

TTS reliability was determined by computing both the Intraclass Correlation Coefficient (ICC) and the Standard Error of the Measurement (SEM). These values were computed using the mean TTS of the odd and even trials. The ICC was computed by an absolute agreement two-way random model because: a) every individual provided scores for all trials (absolute agreement), b) it considers both random and systematic error (two-way) and c) even all the levels of the factor in the design are included, only a sample of the possible levels, and the analysis are used to generalize the results to other levels (random model). ICC was obtained following analytical method by Weir [23]. Values <0.2 (negative values included) were considered as poor, between 0.2 to 0.4 as fair, between 0.4–0.6 as moderate, from 0.6–0.8 as substantial and > 0.8 as good [24]. SEM was obtained using Eq (1) [23].

$$SEM=SD\cdot \sqrt{\left(1-ICC\right)}$$ Eq (1)

In order to compare the TTS values and the reliability of the different signal processes we obtained the best threshold for each one by computing the mean of the height and GRF axis ICC values for each signal type and threshold. Thresholds that provided TTS values lower than 1 s (approximately the minimum value cited in the literature [12]) or using less than 85% of the signals (more than 0.15 parts per unit of unusable trials) were discarded. The best threshold of each method was the one with the highest ICC value.

Finally, the sensibility to detect changes in TTS of the different methods used was established. For that, the effect of the drop jump height on the TTS was established for each axis and computed method independently using several repeated measures ANOVAs. Bonferroni correction was used to correct for multiple effects (i.e., since three axis were included the alpha level was multiplied by 3). When significant effects were found, pairwise comparisons with Bonferroni correction were requested. Eta partial square and Cohen’s d were computed as effect size. The level of significance was set at p = 0.05 for all the analyses.

## Results

**Fig 2:**
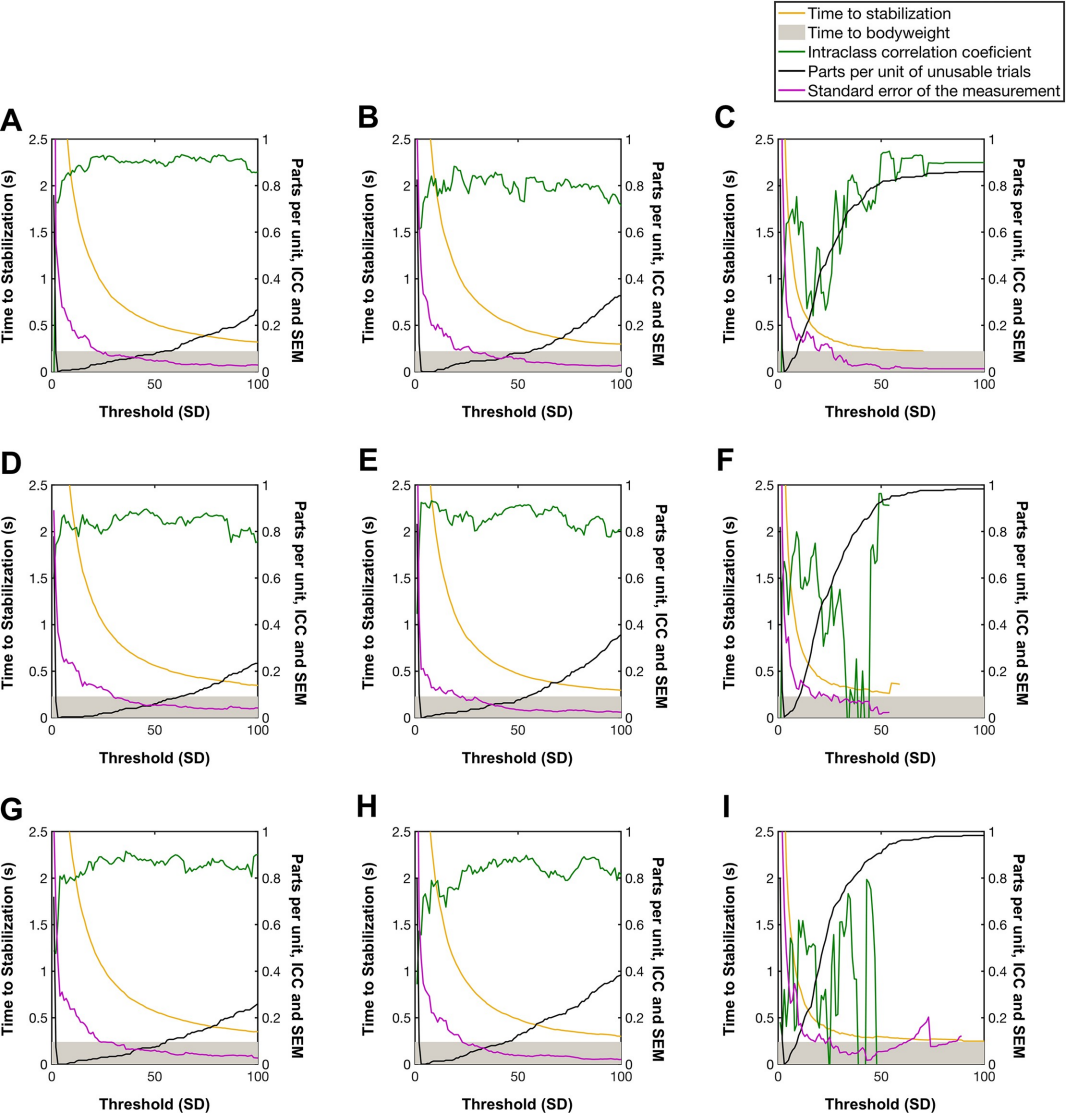
Reliability of the time to stabilization values in function of the threshold used. A: vertical axis and 15 cm height, B = antero-posterior axis and 15 cm height, C = medio-lateral axis and 15 cm height, D: vertical axis and 20 cm height, E = antero-posterior axis and 20 cm height, F = medio-lateral axis and 20 cm height, G: vertical axis and 25 cm height, H = antero-posterior axis and 25 cm height, I = medio-lateral axis and 25 cm height.

Fig 2 shows the influence of the threshold on the ICC, SEM, TTS and parts per unit of unusable trials using the WAV signal. As can be seen, the ICC was relatively high for all the heights both in vertical GRF (Fig 2A, 2D and 2G) and antero-posterior GRF (Fig 2B, 2E and 2H). There were relatively few unusable trials in thresholds between 5 to 50 SDs. In this period the TTS decreased as the threshold level increased (apparently following an exponential function). However, for the medio-lateral GRF (Fig 2C, 2F & 2I) the range of thresholds in which the unusable trials were under 15% was between 3 to 10 SDs. In this range the ICC value was fair to moderate. The rest of the signals (SA, TOP, RMS and RAW) used are reported in supplementary material. Overall, the ICC values were lower and the threshold level had a greater influence on them than with the WAV signal.

**Fig 3:**
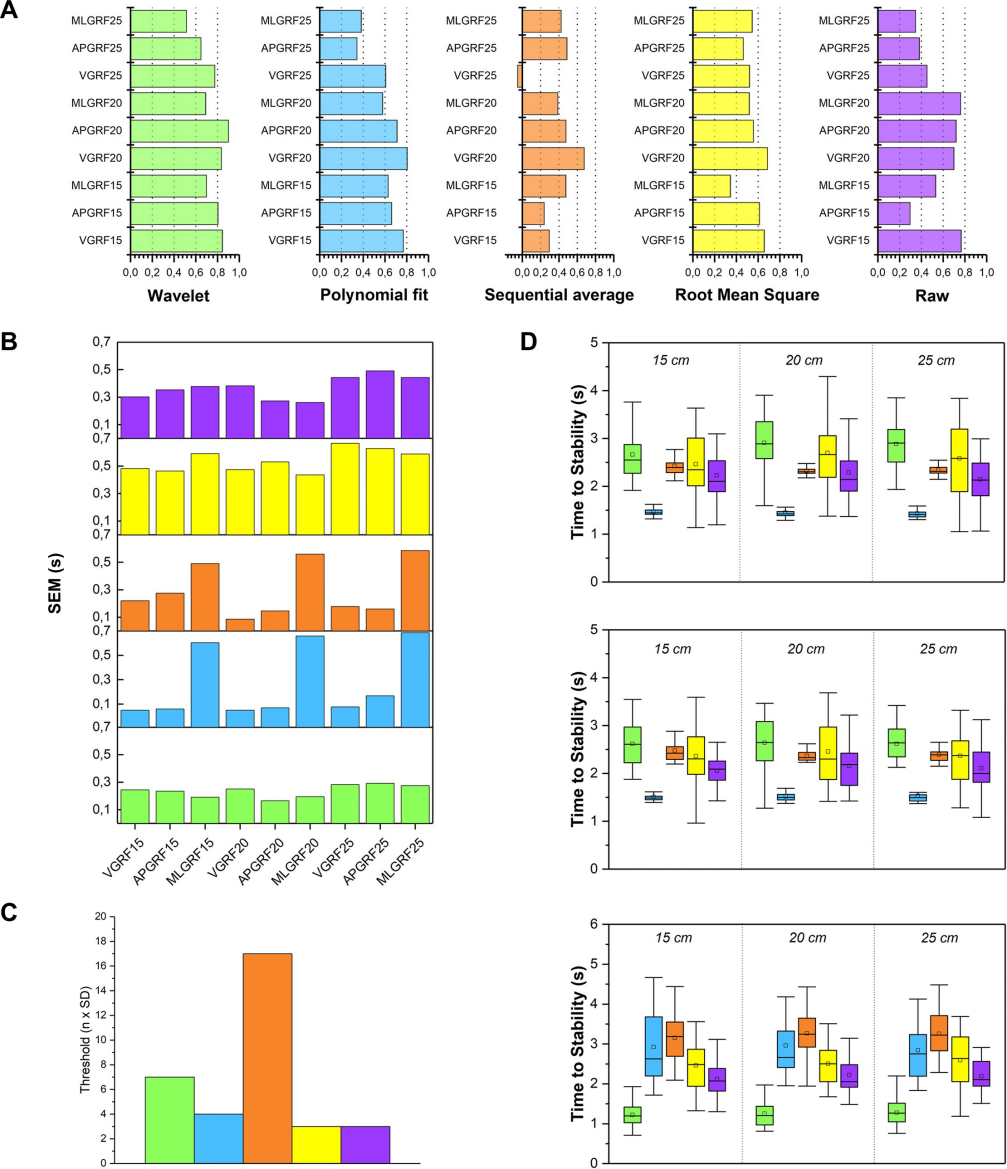
Reliability indexes and time to stabilization values obtained with the five signal processing used. A: intraclass correlation coefficients. B: standard error of the measurement. C: threshold used for each signal used. D: time to stabilization values. MLGRF25 = medio-lateral axis and 25 cm height; MLGRF20 = medio-lateral axis and 20 cm height; MLGRF15 = medio-lateral axis and 15 cm height; APGRF25 = antero-posterior axis and 25 cm height; APGRF20 = antero-posterior axis and 20 cm height; APGRF15 = antero-posterior axis and 15 cm height; VGRF25 = vertical axis and 25 cm height; VGRF20 = = vertical axis and 20 cm height; VGRF15 = = vertical axis and 15 cm height.

Although there was no clear peak in the ICC, using the proposed procedure we determined the ‘best’ threshold for each signal processing in the terms described in the data analysis section. Fig 3 gives the ICC (A) and SEM (B) of each height and axis using the best threshold for each signal processing (C) as well as the boxplot of the TTS obtained for each one (D). The best ICC values (Fig 3A) were obtained using the WAV signal, followed by the TOP signal, when the SEM was lower and more stable (see Fig 3B). The SEM of the TOP signal gave very low values for V- and AP- GRF but were quite high for the ML-axis.

The TTS values obtained for each height, direction and signal (Fig 3D) with the WAV signal were similar to those obtained by SA, RMS and RAW signals for both the GRF in V- and AP-axis (for all jump heights), although the values obtained for the ML-axis were relatively small.

Finally, there was found a significant effect of the drop jump height on the TTS computed with WAV (F_2,58_ = 8.45; p = 0.003; η_p_^2^ = 0.23) and SA (F_2,58_ = 7.12; p = 0.009; η_p_^2^ = 0.2) methods in the vertical direction. Using the WAV method, the TTS was greater in 20 cm and 25 cm than in 15 cm drop jumps (p = 0.001; d = 0.43 and p = 0.009; d = 0.39, respectively). Unexpectedly, TTS computed with SA method reported higher values in 15 cm drop jumps than in 20 and 25 cm ones (p = 0.016; d = -0.6 and p = 0.025; d = 0.5, respectively). Descriptive statistics are reported in Table 1.

**Table 1:** Pairwise comparisons between drop jumps heights.

Method	Axis	Height		
		15 cm	20 cm	25 cm
**Wavelet**	Vertical	2.66 (0.58)	2.91 (0.57) *	2.88 (0.54) *
	Antero-Posterior	2.62 (0.48)	2.64 (0.50)	2.62 (0.42)
	Medio-Lateral	1.22 (0.30)	1.25 (0.31)	1.27 (0.32)
**Polynomial fit**	Vertical	1.47 (0.09)	1.45 (0.10)	1.43 (0.11)
	Antero-Posterior	1.51 (0.09)	1.52 (0.12)	1.53 (0.18)
	Medio-Lateral	2.92 (0.85)	2.96 (0.85)	2.84 (0.71)
**Sequential average**	Vertical	2.42 (0.20)	2.32 (0.13) *	2.34 (0.12) *
	Antero-Posterior	2.48 (0.24)	2.36 (0.17)	2.39 (0.19)
	Medio-Lateral	3.15 (0.55)	3.27 (0.56)	3.26 (0.61)
**Root mean square**	Vertical	2.46 (0.71)	2.70 (0.74)	2.58 (0.79)
	Antero-Posterior	2.36 (0.64)	2.46 (0.66)	2.36 (0.69)
	Medio-Lateral	2.46 (0.56)	2.50 (0.52)	2.59 (0.72)
**Raw**	Vertical	2.23 (0.56)	2.29 (0.61)	2.14 (0.48)
	Antero-Posterior	2.05 (0.32)	2.15 (0.46)	2.11 (0.49)
	Medio-Lateral	2.12 (0.45)	2.22 (0.48)	2.19 (0.44)

Data are expressed as mean (standard deviation).

* Indicates significant differences regarding 15 cm height (p < 0.05).

## Discussion

The main contribution of this study is the proposed new signal processing system to compute TTS in children after a single leg drop jump landing. All of the four signal processing systems used for this purpose until now analyze the signal in the time domain. Our new approach goes one step further by analyzing the signal in a time-scale (frequency) domain by means of continuous wavelet transformation. The time-frequency domain’s principle advantage is its double resolution not only the time but also the frequency domain [21]. An automatic computational method was also developed to establish the ‘best’ threshold for each of the five signal processing systems used. The most noteworthy finding is that the new method to obtain TTS (i.e., WAV) provides the higher reliability indexes and was the most sensitive one to detect changes according to the height of the jump.

Fransz et al. [14,15] recently reported that there were no thresholds that clearly provide the best reliability values in any of the signal processing systems used in their study (RAW, RMS, SA and TOP). However, this paper proposes a method of finding a threshold for each of the systems tested for use in future work on children. The method used is simple and has three main requirements: the first is that the same threshold should be used for all the axes and heights, the second is that it should allow the TTS to be calculated in at least 85% of cases (i.e. only 0.15 parts per unit of unusable trials) and the third that the mean TTS of the subjects should be at least 1 s (based on previously published data [12]). This simple solution provides a threshold for each signal processing system, with substantial-to-good reliability and facilitates decision making on this issue.

Fransz et al. [15] have also suggested that in studies on children SA and TOP are sufficiently reliable in the V- and AP-axis, but not in the ML-axis. In young adults, Flanagan, Ebben and Jensen [25] found an ICC value of 0.42 and 0.68 for single and average measures, respectively, using the RAW signal only in the V-axis., indicating that better reliability indices could be obtained. As expected, we found some fairly good ICC values using SA and TOP in the ML-axis GRF ML-axis in 25 cm and 20 cm drop jumps for TOP and SA, respectively. Additionally, the new WAV signal processing system obtained substantial-to-good ICC values with children for most heights and axes (with the exception of the ML-axis in drop jumps from 25 cm, in which the ICC≈0.5 was only moderate). The SEM values for the WAV system remained stable between 0.1 to 0.2 s, indicating that this system could improve TTS reliability in children. It should also be noted that although this system needs more computational time than those previously used, current devices can perform the analysis in a few seconds. Similar procedures have been used to analyze other kind of digital signals [20,21].

Finally, it would seem that signal processing reliability depends on the height of the drop jump, i.e., the higher the jump the lower the reliability indices. This can be seen in the lower ICC values (GRF ML-, AP- and V-axis from 25 cm in Fig 1A) of the highest jumps, especially in the WAV, TOP and RAW signals. However, Fransz et al. [14] suggest that height does not affect threshold reliability in adolescents because variability increases with height both between and within subjects, although in our opinion the higher variability reduces the ICC, as our values indicate that the higher the drop jump height the lower the ICC value. Future work should explore this topic in greater depth to corroborate this finding, not only in children but also in adolescents and adults.

This study has certain limitations that should be taken into account. Firstly, despite in the current study the sample size estimation confirmed it is a good enough sample, this could be seen as relatively small limiting generalization of the results to other child populations. Secondly, although we tested the effect of height on the reliability index, since lower reliability was found in 25 cm than in 15-to-20 cm jumps, future work should explore how the thresholds are affected from greater heights.

As the WAV method used to process GRF signals provided the highest reliability indices in computing TTS, we here propose an auto-threshold selection system that would allow researchers to fix the threshold of the recommended SD number, according to the processing signal used (seven SDs for WAV processing). These signal processing and threshold levels provide substantial-to-good ICC values and low SEM for almost all axes and heights. The results thus suggest that the WAV method can be used to compute overall TTS to analyze dynamic postural stability in children.
